# Preparation and study of chemical, sensory, and nutritional values of balanced polyunsaturated fatty acid safflower seed oil blended oil

**DOI:** 10.1016/j.fochx.2025.103187

**Published:** 2025-10-17

**Authors:** Xiaochun Zheng, Gaoqian Zhang, Kejun Wei, Hongbin Wu, Jinhu Tian, Xinwen Xu, Wenyu Liu, Min Liu, Changqing Wei

**Affiliations:** a Xinjiang Academy of Agricultural and Reclamation Science, Xinjiang Uygur Autonomous Region, PR China.; b Key Laboratory of Agricultural Product Processing and Quality Control of Specialty (Co-construction by Ministry and Province), School of Food Science and Technology, Shihezi University, Shihezi 832000, Xinjiang Uygur Autonomous Region, PR China.; c Nutrition and Safety Control of Xinjiang Production and Construction Corps, School of Food Science and Technology, Shihezi University, Shihezi 832000, Xinjiang Uygur Autonomous Region, PR China.; d Engineering Research Center of Storage and Processing of Xinjiang Characteristic Fruits and Vegetables, Ministry of Education, School of Food Science and Technology, Shihezi University, Shihezi 832000, Xinjiang Uygur Autonomous Region, PR China.; e Binzhou Zhongyu Food Company Limited Key Laboratory of Wheat Processing, Ministry of Agriculture and Rural Affairs National Industry Technical Innovation Center for Wheat Processing, Shandong Province 256600, China.; f Cooperation Base of Health Food Manufacturing and Quality Control, Zhejiang University, Hangzhou 310058, Zhejiang Province, PR China.; g Ili Yaqina Agricultural Development Co. Ili Kazakh Autonomous Prefecture in Xinjiang, 835000, Xinjiang Uygur Autonomous Region, PR China.; h Key Laboratory of Xinjiang Phytomedicine Resource and Utilization of Ministry of Education, Shihezi University, Shihezi 832000, Xinjiang Uygur Autonomous Region, PR China.; i Institute for Safflower Industry Research of Shihezi University, Shihezi University, Shihezi 832000, Xinjiang Uygur Autonomous Region, PR China.

**Keywords:** Blended cold pressing, Polyunsaturated fatty acids, Nutritional value, Chemical sensory properties, Aroma compounds

## Abstract

Balanced polyunsaturated fatty acids (PUFAs) ratio, nutritional value and flavor are key edible oil attributes. Based on pressure technology, mixing ratio equations, response surface methodology, and analyses of physicochemical and nutritional indexes as well as key volatiles, this study explored oilseed mixing ratio, optimal extraction conditions in the blended cold pressing (BCP) process, and blended oil's physicochemical properties, nutritional profile, and aroma. Results showed the optimal flaxseed:safflower seed mass ratio was 1:(1.66–2.6); best extraction conditions: 64 °C pressing temperature, 6.7 % moisture, 1.5-min 800 W microwave treatment, with a final oil yield of 19.83 %. BCP oil (BCPO) showed enhanced oxidative stability, improved nutritional functions, and richer volatiles (aldehydes, alcohols, pyrazines). Sensory evaluation showed BCPO had prominent vegetable, roasted and overall flavors. This study developed a one-step BCP process to prepare blended oils with balanced PUFAs, providing a theoretical basis for BCP application in edible oil processing.

## Highlights

• Microwave cold pressing effectively increased the oil yield of oilseeds.
• Extraction conditions in the blended cold pressing process were optimized.
• Nutritional properties of BCPO were enhanced.
• 17 key aromatic compounds were identified in BCPO.
• The BCP process has some advantages in terms of aroma enhancement.

## 1 Introduction

Polyunsaturated fatty acids (PUFAs), recognized as essential nutrients, are crucial in regulating blood lipid metabolism and boosting immune function within the diet. Among these, n-3 and n-6 PUFAs are particularly important due to their significant effects (Schneedorferová et al., 2015). In the human system, long-chain fatty acids can be synthesized from two PUFA precursors: α-linolenic acid (ALA) serves as the precursor for n-3 PUFAs, while linoleic acid (LA) is used for n-6 PUFAs (Adkins & Kelley, 2010; Simopoulos, 1991). Current nutritional research is increasingly focused on the intake of appropriate proportions of PUFAs. The Chinese Dietary Reference Intakes (Chinese DRIs) indicate that the suggested ratio for the intake of n-6 to n-3 PUFA is between 4:1 and 6:1. The dietary habits of the general public are currently marked by an inadequate consumption of n-3 PUFAs and an excessive consumption of n-6 PUFAs. Research has shown that a high n-6/n-3 PUFA ratio in the diet can negatively impact human health by promoting the production of pro-inflammatory cytokines, such as interleukin-1, thereby exacerbating inflammation (Simopoulos, 2002).

Vegetable oils are one of the main sources of human intake of PUFAs, and safflower oil (SO) is commonly utilized in the food sector due to its significant traits of high polyunsaturation (Conte et al., 2016). It is known that SO is recognized for its high content of C18-based n-6 PUFAs, particularly LA, which has the potential to lower blood cholesterol levels (Wilson et al., 2006). However, the proportion of n-6/n-3 PUFA in SO does not entirely align with the criteria set by the Chinese DRIs. Currently, flaxseed oil (FO) is frequently utilized in manufacturing to control the n-6/n-3 PUFA ratio, enabling the creation of functional products, thanks to its substantial ALA (n-3 PUFA) content (Li et al., 2022). Research indicates that alpha-linolenic acid (ALA) can be metabolized into docosahexaenoic acid (DHA) and eicosapentaenoic acid (EPA) within the body, which can contribute to reductions in blood pressure, the prevention of blood clots, and anti-aging effects (Derbali et al., 2015). Therefore, blending FO with SO helps balance saturated and monounsaturated fatty acids (MUFAs), meeting the need for edible oil with properly proportioned PUFAs.

Hot pressing and cold pressing, collectively known as physical pressing, are the common methods of oil extraction used in the food industry today. Compared with hot pressing, cold pressing largely overcomes the problem of quality degradation in oils caused by the high-temperature denaturation of the protein during the pressing process, and a variety of natural active substances such as phytosterols, tocopherols, and other ingredients are retained (Teh & Birch, 2013). Nevertheless, there are some problems with cold-pressed oil, mainly yield and flavor. Oil yield is a significant economic indicator in the production of oil processing, and volatile aroma substances are important indicators for evaluating the quality of vegetable oils (He et al., 2021). To solve these problems, the oilseeds can be pretreated with a microwave. Many studies have found that microwave pretreatment of oilseeds before extraction can not only improve oil yields, but the aroma in the oils will be more intense (Suri et al., 2020; Zhou et al., 2013). A blending of vegetable oils to adjust the fatty acid ratio is a frequently used method, but this method of blending after pressing may lead to a loss of oils and antioxidants. The blended cold pressing (BCP) process is a method of pressing in which a variety of oilseeds are blended in a certain proportion before being pressed. This method not only continues many of the advantages of cold pressing, but also avoids the loss of oil and antioxidant substances due to secondary processing.

Information on the process of preparing PUFAs equilibrium oil by BCP is scarce, and the characteristics and differences in the nutritional value and aroma sensory characteristics of oils produced under BCP process conditions compared to oil products blended after cold pressing are also unclear. In this study, the blending ratios of safflower seed and flaxseed were determined based on the oil yields, fatty acid compositions and recommended dietary intake, and the effects of moisture content, microwave treatment time, and pressing temperature on the PUFAs equilibrium oil yield were investigated by response surface methodology (RSM) to obtain the optimal BCP conditions. Furthermore, basic physico-chemical, nutritional indexes and key flavor substances of blended cold pressed oil (BCPO), the blended oil after cold pressing (BO), and SO under optimal conditions were tested and analyzed. Finally, their sensory evaluation was conducted. This research aims to establish a theoretical foundation for the generation of PUFAs equilibrium oil to satisfy balanced nutritional needs, in addition to offering a practical reference for the BCP process within the realm of oil studies.

## 2 Materials and methods

### 2.1 Materials

Both safflower seeds and flaxseeds were purchased at the local market in Shihezi, China, with a moisture content of 4.6 %. The purchased seeds were treated using the method reported by a previous study (Musa Özcan et al., 2019). The seeds were cleaned by washing and screening out straw, crushed stones, and other impurities, then poured into polypropylene bags and kept in a cool, dry environment for further use. Reagents used in this study were acquired from Tianjin Fuyu Fine Chemical Co. (Tianjin, China), and n-heptane and high-performance liquid chromatography (HPLC)-grade methanol for gas chromatography (GC) were purchased from Fisher Chemical (Ottawa, Canada). The standards of palmitic acid, stearic acid, oleic acid, linoleic acid, linolenic acid and tocopherol were supplied by Sigma–Aldrich Inc. (St. Louis, MO). Other reagents employed in this study were of analytical grade.

### 2.2 Determination of oilseed mixture proportion

#### 2.2.1 Pretreatment of safflower oil and flaxseed oil

Each 300 g oilseed sample in a porcelain plate was roasted in a microwave oven (Midea Group Co., Ltd., M1-L201B, Guangzhou, China) at 800 W for 2 min (pre-experiment determined). Oilseed samples were cooled to room temperature and ready to be pressed. Additionally, the moisture content of the samples changed minimally (from 4.6 % to 4.5 %) after 2 min of microwave treatment.

The oilseed samples were subjected to cold pressing (CP), microwave cold pressing (MW-CP), and hot pressing (HP), respectively. Treated and untreated oilseeds were both pressed by a BGC-T15 screw oil press (Dongguan Electric Appliance Co., Ltd., Dongguan, China) at 65 °C to obtain crude oils. Meanwhile, the untreated oilseeds were processed by hot pressing at 140 °C. After their extraction, the crude oils underwent filtration and were centrifuged at 6500 rpm for 15 min. The safflower oil (SO) and flaxseed oil (FO) were ultimately kept in sealed dark-colored bottles and stored at 4 °C for subsequent use (Musa Özcan et al., 2019).

#### 2.2.2 Determination of oil yield

The calculation of oil yield (OY) refers to the method described in previously published articles (Perrier et al., 2017; Zhang et al., 2017). Specifically, the OY of different oilseed seeds was calculated by the following formula:

(1) $$\mathrm{OY}\;\left(\%\right)=\mathrm{Mo}/\mathrm{Ms}\times 100$$

Mo: mass of oil extracted from oilseeds (g); Ms.: mass of oilseed sample (g).

#### 2.2.3 Determination of fatty acid composition

The fatty acids (FAs) in various oils were determined by GC following the reported method (Sun et al., 2021). Fatty acid methylation was performed in accordance with the Chinese national standard GB 5009.168-2016. Briefly, a 300 mg oil sample was placed in a stoppered test tube, to which 20 mL n-hexane and 8 mL 2 % sodium hydroxide-methanol solution were added. The mixture was refluxed at 80 °C under condensation until droplets vanished. Then a 7 mL 15 % boron trifluoride-methanol solution was added; after 2 min hydrolysis, it was cooled, followed by the addition of 20 mL n-hexane. After vigorous shaking, a saturated sodium chloride solution was added. After standing for 5–10 min, a 5 mL supernatant was taken, dried with anhydrous sodium sulfate, filtered through a 0.22 μm filter, and transferred to a sample vial for GC analysis.

A capillary HP-innowax column (30 m × 0.25 mm × 0.25 μm, HP-5 ms) from Agilent (Palo Alto, CA) was used to perform the chromatographic separation of FAMEs. Nitrogen functioned as the carrier gas, while the samples were introduced into a gas chromatography system (Shimadzu GC-2014) equipped with a flame ionization detector (FID). GC conditions: FID temperature 300 °C; inlet temperature 210 °C; column temperature 180 °C; nitrogen flow rate 1.5 mL/min; hydrogen flow rate 30 mL/min; air flow rate 450 mL/min; make-up gas (nitrogen) flow rate 30 mL/min; injection volume 1 μL.

The FAs content (μg/g) in the refined oil was determined using the standard curve method. Specifically, mixed standard solutions of five FA standards (LA, ALA, OA, SA, and PA) at varying concentrations (0.5, 2, 5, 10, 25 μg/mL) were prepared from their respective stock solutions (1.0 mg/mL), followed by GC analysis. Standard curves were generated via regression analysis (Table S1).

#### 2.2.4 Mass ratio method

The mass ratio method refers to mixing two processed oilseeds (SO/FO, Ws/Wf) at different mass ratios. Each 500 g mixture was processed through microwave-assisted cold pressing to obtain refined oil, and the fatty acid content was determined. By adjusting the mass ratio of microwave-treated safflower seeds to flaxseeds in a certain gradient, the appropriate raw material ratio (a-b) that would ensure the “n-6/n-3 PUFAs” ratio in the BCPO would fall within the recommended range is finally determined.

#### 2.2.5 Fatty acid ratio method

The FA ratio method takes the OY of safflower seeds and flaxseeds, as well as the contents of n-3 and n-6 PUFAs in SO and FO, as parameters. With the recommended intake ratio of PUFAs as the target, the reasonable proportion range (a-b) of safflower seeds to flaxseeds was obtained according to the formula and relevant parameters. The oilseed mixing ratio formula was as follows:

(2) $$n-6\;\text{PUFAs}/n-3\;\text{PUFAs}=\frac{\mathrm{Ws}\times \mathrm{OYs}\times C1+\mathrm{Wf}\times \mathrm{OYf}\times C2}{\mathrm{Wf}\times \mathrm{OYf}\times C3+\mathrm{Ws}\times \mathrm{OYs}\times C4}=4∼6:1$$

Ws: weight of SO after treatment (g); Wf: weight of flaxseeds after treatment (g); OYs: oil yield of treated safflower seeds (%); OYf: oil yield of treated flaxseeds (%); C1: the absolute content of LA in SO (μg/g); C2: the absolute content of LA in FO (μg/g); C3: the absolute content of ALA in FO (μg/g); C4: the absolute content of ALA in SO (μg/g).

### 2.3 Verification of oilseed mixing ratio

In formula (2) of the fatty acid ratio method, the “oil extraction rate” was determined from separate pressing of safflower seeds and flaxseeds. However, this parameter may be affected by oilseed interactions during microwave-assisted mixed cold pressing, so the Ws/Wf determined this way requires verification. Oilseeds were mixed at different ratios (Ws/Wf = 0.00, 0.20, 0.40, 0.60, 0.80, 1.00, 1.20, 1.40, 1.60, 1.66, 1.80, 2.00, 2.40, 2.60, and 2.80). The oilseeds were blended in the calculated proportions and then subjected to microwave treatment, cooling treatment, cold pressing, and determination of FAs content. After pressing, FAs in the crude oil were analyzed as in Section 2.2.4. The accuracy of the blending ratios of the oilseeds was verified by comparing detected “n-6/n-3 PUFAs” ratios with predicted values (from formula (2) and related parameters).

### 2.4 Single-factor experiments on microwave-assisted mixed cold pressing process

#### 2.4.1 Microwave processing time

Safflower and flaxseeds were mixed at a fixed ratio (Ws/Wf = 2), 500 g per group. While other conditions remained unchanged, the mixture was microwave-treated at 800 W for 0, 0.5, 1, 1.5, 2, 2.5, 3 min. Using OY as the index, the optimal time was determined.

#### 2.4.2 Moisture content

Pretreatment was consistent with Section 2.4.1. With other conditions unchanged, using OY as the index, the moisture content of the safflower-flaxseed mixture was adjusted to 2 %, 3 %, 4 %, 5 %, 6 %, 7 %, and 8 % to determine the optimal moisture content.

#### 2.4.3 Cold pressing temperature

The temperature during the mixed cold pressing of the safflower-flaxseed mixture was adjusted to 45, 50, 55, 60, 65, and 70 °C, and OY was used as the index to determine the optimal cold pressing temperature.

#### 2.4.4 Cold pressing time

Using the OY as the index, with other conditions unchanged, the cold pressing time of the safflower-flaxseed mixture was adjusted to 20, 40, 60, 80, and 100 min to determine the optimal cold pressing duration.

### 2.5 Response surface optimization experiment

The effects of independent variables (X1-microwave treatment time, X2-cold pressing temperature, and X3-blended oilseed moisture content) on the dependent variable (Y-oil yield of blended oilseed) were investigated using response surface methodology (RSM), and a three-level, three-factor Box-Behnken experimental design (BBD) was applied to determine the effect of BCP process parameters on the OY (Table S2). The center point and the range of values for each factor were determined based on literature about oil extraction (Delfan-Hosseini et al., 2017) and preliminary experiments via single-factor design.

To evaluate and optimize the effect of process parameters on response variables, the complete design included 17 randomized trials, of which there were 5 replications at the center. To reduce the impact of unforeseen variability in the obtained responses, the experiments were carried out randomly, and the response data were mean values (Homayoonfal et al., 2015). In the experimental design, the yield of blended oilseed (Y) was represented as a function of the independent variable through a second-order polynomial equation, expressed as follows:

(3) $$Y=\beta_{0}+\sum_{n=1}^{3}\beta_{n}X_{n}+\sum_{n=1}^{3}\beta_{\mathrm{nn}}X_{n}^{2}+\sum_{n=1}^{2}\sum_{m=n+1}^{3}\beta_{\mathrm{nm}}X_{n}X_{m}$$

β_0_: the response value at the center point; β_n_, β_nn_, and β_nm:_ the linear, quadratic, and interaction terms, respectively. The design expert software, version 8.0.6, was utilized to ascertain these coefficients.

### 2.6 Preparation of SO blended oil

After adjusting the parameters of BCP according to the optimal conditions optimized by RSM, three sets of samples were prepared: 1) The first set was the PUFAs equilibrium oil obtained by BCP (BCPO); 2) The second set was the blended oil of SO and FO, which were cold-pressed separately and then blended (BO); 3) The third set was cold-pressed SO because safflower seed was the main ingredient of the PUFAs equilibrium oil. It was noted that the three sets of samples were kept the same in all parameters (including the weight, weight ratio of safflower seeds to flaxseeds, filtration and centrifugation parameters, etc.) except for the differences in the blending or pressing methods.

### 2.7 Determination of physical and chemical properties

#### 2.7.1 Basic physicochemical properties

The acid value (AV) was determined referring to the hot ethanol method specified in GB 5009.229-2016. The peroxide value (POV) was measured via the sodium thiosulfate titration method in GB 5009.227-2016. The 2-thiobarbituric acid value (TBA) was determined according to GB/T 35252-2017. The p-anisidine value (p-AV) was measured with reference to GB/T 24304-2009, and the iodine value was determined in accordance with GB/T 5532-2008. The total oxidation value was determined by calculation formula was as follows:

(4) Total oxidation value=2POV+p−AV

#### 2.7.2 Oxidation stability

The oxidation stability of the oils was evaluated by determining their oxidation induction time (in accordance with GB/T 21121-2007) via controlling temperature and air flow. Conditions: heating at 110 °C, with an air flow of 20 L/h. Test tubes were soaked in 3 % strong alkali for 3 h, rinsed successively with distilled water and propanol, and then dried at 80 °C. Electrodes, connecting tubes, and measuring containers were rinsed with alcohol and distilled water, then dried with a nitrogen blower before use.

Accelerated oxidation of the three vegetable oils was tested by Schaal oven method: 180 mL of each oil was dispensed into dark lidded bottles (30 mL/bottle), and heated in an oven at 63 ± 0.5 °C in the dark for 28 d. Samples were taken on days 0, 3, 7, 14, 21 and 28 to determine POV and P-AV.

### 2.8 Determination of FAs, tocopherols and phytosterols

#### 2.8.1 FAs and tocopherols

The method for determining FAs in oil samples was the same as in Section 2.2.3. The tocopherol determination method was slightly modified with previous reported (Abd El-Baset et al., 2024; Hao et al., 2025). Briefly, 2 g of an oil sample was accurately weighed into a 25 mL brown volumetric flask, dissolved and diluted to volume with n-hexane, mixed well, filtered through a 0.2 μm membrane, and the filtrate was stored in a brown bottle for subsequent testing. LC conditions: Shlm-pack C18 column (5 μm, 4.6 × 250 mm), mobile phase of methanol, flow rate 0.8 mL/min, column temperature 20 °C, injection volume 10 μL, detection wavelength 294 nm. Qualitative and quantitative analysis was performed by the external standard method. Stock solutions of α, β, γ, and δ tocopherol (1.0 mg/mL) were diluted with chromatographic grade methanol to prepare standard solutions of various concentrations (0.2, 0.5, 1, 2, 4, 6, 8, 10, 50, 100 μg/mL), and standard curves were generated by regression analysis (Table S1).

#### 2.8.2 Phytosterols

The method for the determination of phytosterols in oil samples was slightly modified from Hao et al. (Hao et al., 2025). Briefly, 2 g vegetable oil was mixed with 30 mL 3 mol/L KOH-ethanol solution, saponified at 60 °C for 1 h, and then extracted with n-hexane-water (3:2, *v*/v). The supernatant was dried under nitrogen to get a crude sample, which was dissolved in 2 mL absolute ethanol. Then, 0.1 mL of the solution was pipetted and mixed with 2 mL phosphosulfuric acid‑iron reagent, diluted to 6 mL with absolute ethanol, and the absorbance was measured at 520 nm. Sterol content was calculated via the standard curve. Stigmasterol was used as standard: a 100 μg/mL solution in absolute ethanol was prepared. Aliquots (0.0, 0.5, 1.0, 1.5, 2.0, 2.5 mL) were mixed with 2 mL reagent, diluted to 6 mL, and absorbance at 520 nm was measured to generate the standard curve via regression analysis (Table S1).

### 2.9 Determination of volatile compounds

The oil samples' volatile compounds were extracted and analyzed through headspace gas chromatography–mass spectrometry (HS-GC–MS), following the methods outlined in earlier research (Sun et al., 2021; Tian et al., 2021) with slight modification. In this study, 10.0 g of prepared oil samples (BCPO, BO and SO) was put in a 20 mL headspace vial and then 2.5 μL 3-octanol (internal standard, 8.18 μg/mL in hexane) was added to the headspace vial. Thereafter, every headspace vial was sealed with an aluminum cap and Teflon septum. The carrier gas utilized was high-purity helium, flowing at a rate of 1.5 mL/min. HS-GC–MS analysis was performed on an Agilent Technologies 7890B GC–MS system, and chromatographic separation was achieved using a GC fused silica capillary column (HP-5, 30 m × 0.25 mm × 0.25 μm) from Agilent. The temperature program was as follows: Initially, the temperature was raised to 40 °C and maintained for 2 min. Following this, it was incremented to 110 °C at a rate of 5 °C/min and sustained for 5 min. Afterward, the temperature was increased to 230 °C at a rate of 12 °C/min and held constant for 10 min. The injector temperature was 250 °C and the injection volume was 1 μL in splitless mode. Ionization was performed under 70 eV electron ionization (EI) mode, and spectra were recorded in full scan over the range 30–450 (*m*/*z*). The identification of volatile flavor compounds primarily relied on computer matching with the NIST11 mass spectral library. Quantitative analysis was done by internal standard method. The formula was as follows:

(5) $$\mathrm{Cx}=\frac{S_{X}\times V_{0}\times C_{0}}{M\times S_{0}}$$

C_x_: concentration of the unknown compound (μg/g); M: mass of the oil sample (g); S0 and Sx: peak areas (AU·min) of the internal standard substance and the unknown compound respectively; V_0_: injection volume of the internal standard (μL); C_0_: content of the internal standard (μg/μL).

### 2.10 Odor activity value (OAV) for aroma compounds

The odor activity value (OAV), which represents the ratio between the concentration of a compound and its odor threshold, is frequently utilized to assess how volatile compounds contribute to flavor (Wang, Ma, et al., 2020). The calculation of OAV was based on the following formula:

(6) OAV=C/OT

C: concentration of the volatile compound; OT: published odor threshold (Jia et al., 2019).

### 2.11 Sensory evaluation

Sensory evaluation of BCPO, BO and SO followed He et al.'s method (He et al., 2021). Ten trained panelists (5 males, 5 females, aged 20–30 years) performed descriptive analysis in a 25 °C laboratory with a suitable environment. 5.0 g oil samples in 20 mL glass vials were evaluated for odor descriptors: oiliness, grassiness, roasted aroma, burnt smell, freshness, fishiness, sourness, and overall flavor. The scoring criteria were as follows: extremely strong (8–10), strong (6–8), moderate (4–6), weak (2–4), extremely weak (0–2), non-existent (0). Panelists rinsed their mouths with water between samples.

All participants were informed of the experiment's purpose, signed written informed consent prior to participation, and the sensory evaluation adhered to the Declaration of Helsinki. The privacy of all participants was rigorously protected throughout the investigation. Our institution granted ethical permission to conduct human sensory study. Participants provided informed consent through the statement, ‘I am aware that my responses are confidential, and I agree to participate in this sensory evaluation’, which required affirmative responses to proceed with sensory evaluation. All participants have the ability to withdraw from the study at any time.

### 2.12 Statistical analysis

All experiments were performed in triplicate, and the results were presented as mean ± standard deviation (SD). The experimental data for the response surface model underwent multiple regression analysis to develop a second-order polynomial equation based on the independent variables. Design of experiments, model analysis, and analysis of variance (ANOVA) were conducted using Design Expert 8.0.6 at a 95 % confidence interval (*p* < 0.05). Contour plots and response plots were created to visualize the effect of the independent variables on the response variables. The volatile flavor data of the samples were analyzed using principal component analysis (PCA). Statistical analysis was performed using SPSS Statistics 26, Origin 17, and Microsoft Office Excel 2008.

## 3 Results and discussion

### 3.1 Determination and analysis of oilseed mixing ratio

In preparing PUFA-balanced oil, the oilseed blending ratio is critical as it greatly affects the proportion of unsaturated FAs. This study investigated the effects of different pressing processes on the oil yields of safflower seeds and flaxseeds, as well as the impact of the optimal process on the PUFAs contents of SO and FO, and finally determined the oilseed blending ratio.

#### 3.1.1 OY

**Fig. 1:**
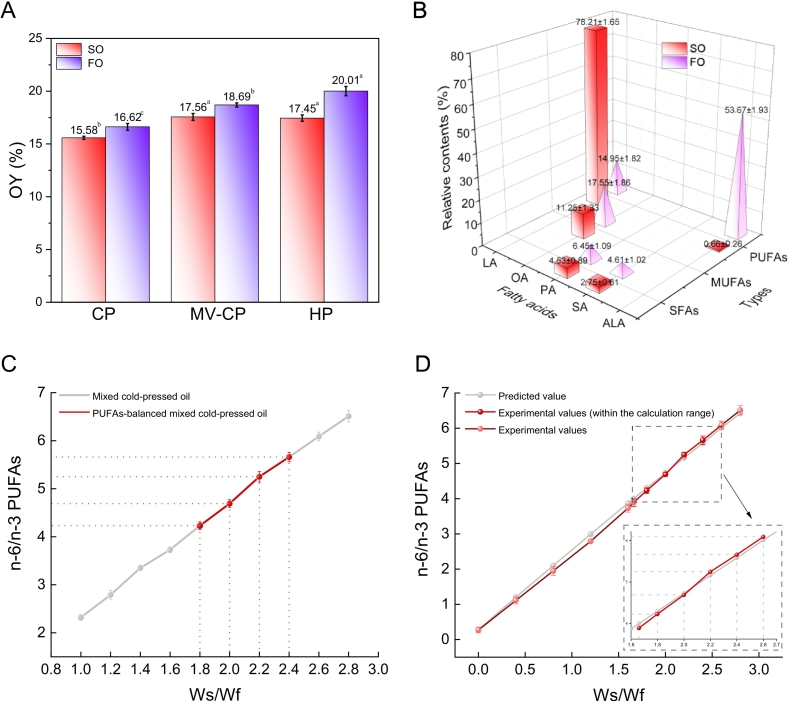
Determination of parameters for SO and FO. A, oil yields of safflower seeds and flaxseeds under different pressing processes. CP, cold press; MV-CP, microwave cold press; HP, hot press. B, fatty acid compositions (%) of SO and FO extracted by MC-CP. SFAs, saturated fatty acids; MUFAs, monounsaturated fatty acids; PUFAs, polyunsaturated fatty acids. LA, linoleic acid; OA, oleic acid; PA, palmitic acid; SA, stearic acid; ALA, α-linolenic acid. C, proportions of n-6/n-3 PUFAs in blended cold pressed oils with different proportioning conditions; D, Verification of n-6/n-3 PUFAs in blended cold pressed oils. The figure compared predicted and experimental n-6/n-3 PUFA values in blended cold-pressed oils with different oilseed ratios. The experimental curve was split into calculated and non-calculated ranges to better reflect and analyze the trend of differences between the two. Values and error bars represented the means and standard deviations of duplicated experiments (*n* = 3), respectively. Different letters in the figure indicated significant differences (*p* ≤ 0.05).

OY in oilseeds is a production indicator that needs to be paid attention to in food industrial processing. Under the CP process, the oil yields of SO and FO were 15.58 ± 0.15 % and 17.62 ± 0.32 %, respectively (Fig. 1A). The difference in the yields of the two oils is mainly related to the composition of the two oilseeds, the proportion of core-shell and plant species, etc. (Silva et al., 2018; Khalid et al., 2017). Compared with the CP process, the oil yields of SO and FO significantly increased (*p <* 0.05) in the MV-CP conditions. After 2 min of microwave treatment at 800w for safflower seed and flaxseed, the oil yields increased to 19.56 ± 0.33 % and 20.69 ± 0.19 %, respectively. Microwave treatment of oilseeds can largely overcome the problem of low oil yield under cold pressing conditions. And it is also worth noting that the main ingredient used in this study, SO, has no significant difference (*p* > 0.05) in oil yield between HP and MV-CP processes. Therefore, microwave-cold pressing could be considered as an efficient and feasible extraction method. Microwave treatment of oilseeds prior to cold pressing has been reported to improve oil extraction yield (Ren et al., 2015), it has been demonstrated that the low oil yield of oilseeds without microwave treatment was due to their intact cell wall during the pressing process, while microwave pretreatment can permanently change the internal cell structure of oilseeds, the cell wall is modified and the porosity is improved, thus improving the oil extraction efficiency (Uquiche et al., 2008).

#### 3.1.2 FAs in SO and FO

SO and FO are considered to be important sources of essential FAs for human intake, especially n-3 PUFAs and n-6 PUFAs. The FAs profiles of SO and FO extracted under MV-CP conditions showed that a high proportion of PUFAs followed by MUFAs and a low proportion of saturated FAs (SFAs) (Fig. 1B). The main FAs, palmitic acid (C16:0, PA), stearic acid (C18:0, SA), oleic acid (C18:1, OA), linoleic acid (C18:2n6c, LA), and α-linolenic acid (C18:3n3c, ALA), were detected in SO and FO. These five FAs accounted for more than 97 % of lipids in both oils. In SO, the majority of lipids in SO were PUFAs (78.87 %), with LA being the most abundant (78.21 %) and ALA being the lowest (0.66 %). Furthermore, the MUFA was mainly OA (11.25 %), and the SFAs were mainly PA and SA (4.53 % and 2.75 % respectively). In FO, the sum of PUFAs, MUFAs, and SFAs accounted for 68.62 %, 17.55 % and 11.06 %, respectively. The ALA (53.67 %) and LA (14.95 %) were the main PUFAs, and the content of ALA was much higher than other types of FAs. In addition, the major MUFA was OA (17.55 %) and the major SFAs were PA (6.45 %) and SA (4.61 %). Specifically, the contents of LA and ALA in SO and FO were determined as follows: 16.38 μg/g (LA in SO), 1.86 μg/g (LA in FO), 0.21 μg/g (ALA in SO), and 6.67 μg/g (ALA in FO). Furthermore, the absolute contents of ALA and LA in FO were much close to the experimental results of previous study (Sun et al., 2021), and the reasons for the differences might be due to the pressing temperature and frying time of FO. Herein, the results of the assay were remarkably close to the reported FAs composition of SO and FO, ensuring the quality and authenticity of the samples tested (da Silva et al., 2018; Khalid et al., 2017; Suri et al., 2020).

#### 3.1.3 Oilseeds blending and ratios verification

To determine the oilseed mass ratio range for BCPO where the n-6/n-3 PUFA ratio meets dietary recommendations, this study explored PUFA proportion ranges via mass ratio and FAs ratio methods. Results from the mass ratio method showed: at Ws/Wf = 1, the n-6/n-3 PUFA ratio was 2.32 ± 0.06, below the recommended level (Fig. 1C). As Ws/Wf increased, SO proportion rose, and the n-6/n-3 ratio grew nearly linearly. When Ws/Wf was 1.8–2.4, PUFA proportions in the blend met dietary recommendations. At Ws/Wf = 2.6, the n-6/n-3 ratio was 6.09 ± 0.09, slightly exceeding the upper recommended limit. Next, the FAs ratio method—based on the oilseed blending formula and parameters—showed the reasonable safflower-to-flaxseed ratio range as 1.66–2.60 (Fig. 1D). To ensure reliability, we verified n-6/n-3 PUFAs in BCPO with safflower-flaxseed mass ratios from 0.00 to 2.80:1 (Fig. 1D). Results showed that when Ws/Wf < 2.0, experimental n-6/n-3 PUFA values in the BCPO were slightly lower than predicted, with their difference first increasing then decreasing. At Ws/Wf = 2.0, the two values were closest. When Ws/Wf > 2.0, experimental values were slightly higher than predicted and stabilized (Fig. 1D). At lower Ws/Wf levels, flaxseeds accounted for a higher proportion in the pressing system, and they contain approximately 10 % flaxseed gum, the main component of flaxseed mucilage. Per Bouallegue et al.'s extraction model, high viscosity hindered safflower seed lipid extraction in microwave-assisted cold pressing, reducing n-6 PUFA content and making experimental n-6/n-3 ratios lower than predicted (Bouallegue et al., 2019). As Ws/Wf rose, safflower seed proportion increased, lowering system viscosity and facilitating lipid extraction of safflower seed. This made experimental values approach predictions and eventually stabilize. Thus, predicted and experimental curves fitted well within the FAs ratio-calculated range, confirming high reliability of the oilseed blending ratios. Overall, the “n-6/n-3 PUFAs” ratio in the BCPO was somewhat away from both the lower and upper limits of the dietary recommendation (Fig. 1C and D). Thus, Ws/Wf was set at 2.0 for convenience and accuracy in subsequent experiments.

### 3.2 Optimization of MV-CP for oil extraction

#### 3.2.1 Influence of different parameter conditions on OY

**Fig. 2:**
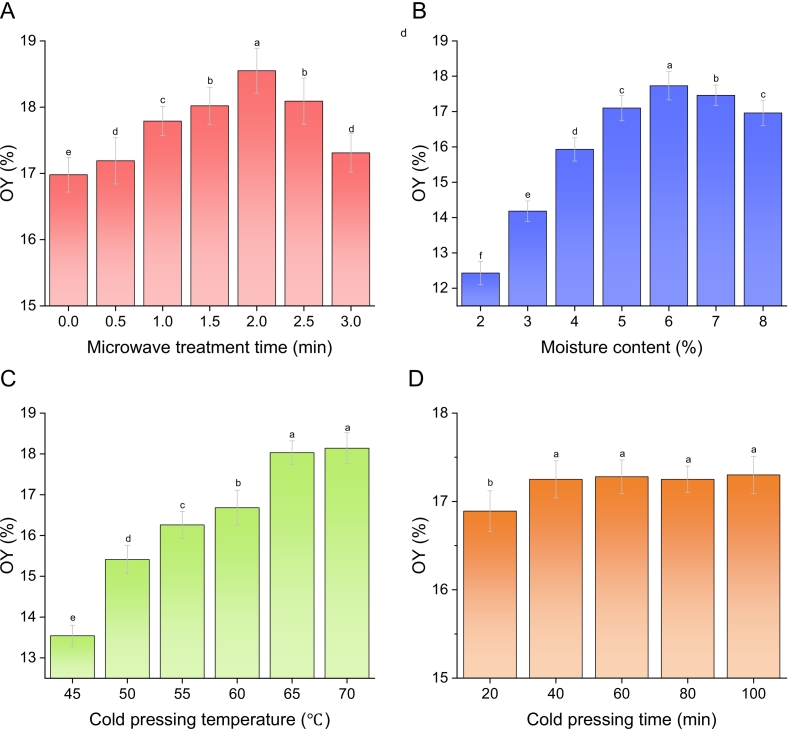
Influence of parameter conditions on OY of BCPO. A, B, C and D represented the effect of microwave treatment time, moisture content of oilseed, cold pressing temperature, and cold pressing time on the OY of BCPO, respectively. OY, oil yield; BCPO, blended cold pressed oil. Values and error bars represented the means and standard deviations of duplicated experiments (n = 3), respectively. Different letters in the figure indicated significant differences (p ≤ 0.05).

Microwave pretreatment can permanently alter oilseed cell structure, improving permeability and porosity (Delfan-Hosseini et al., 2017). However, OY and retention of nutritional functional components of oil are closely linked to microwave time, moisture content, cold-pressing temperature and time. Results showed that the OY of mixed oilseeds without microwave treatment was 16.98 ± 0.26 % (Fig. 2A). It increased with microwave time, peaking at 18.55 ± 0.34 % at 2 min, then decreased. At 2 % moisture, the OY of mixed oilseeds was 12.43 ± 0.33 % (Fig. 2B). It rose with higher moisture, peaking at 17.73 ± 0.4 % at 6 %, then declined. At 45 °C CP, the OY was low (13.54 ± 0.26 %) (Fig. 2C). It rose with temperature, stabilizing at around 18 % when the pressing temperature reached 65–70 °C. Additionally, when the CP time was set to 20 min, the OY was 16.89 ± 0.23 % (Fig. 2D). As the CP time prolonged, the growth rate of the OY tended to slow down. When the CP time was extended beyond 40 min, there was no significant change in the OY (*p* > 0.05). In general, microwave treatment time, moisture content, and pressing temperature of oilseeds had an important effect on OY during the cold pressing process, while the effect of pressing time was relatively small. Microwave treatment can destroy the cell structure of oilseeds. In a certain range, with the increase of microwave treatment time, the absorbed energy in the oilseeds is higher and higher, and the internal molecular vibration is strengthened, which is conducive to improving the oil yield (Lokman et al., 2015). For moisture content, moderately increasing the moisture content of oilseeds can improve oil yield. This is because the moisture content affects the plasticity of the oilseeds and as the plasticity increases, the oil yield also increases (Kabutey et al., 2025). Nevertheless, too high moisture content and too long microwave treatment time could make the internal energy of oilseeds too high, which may easily cause scorching of oilseeds and is not conducive to production (Ye et al., 2021). Regarding cold-pressing temperature: oilseeds had low binding capacity at low temperatures. As temperature gradually rose, it increased the molecular kinetic energy of the oilseed system, enhancing their binding capacity and plasticity, thereby promoting lipid extraction (Cui et al., 2025). However, higher temperatures also boosted the fluidity of oilseeds in the pressing system, which instead hindered lipid extraction. Under the interactive influence of temperature on the binding capacity, plasticity and fluidity of oilseeds, the growth of the oil extraction rate slowed down and tended to stabilize. Overall, subsequent research parameters: microwave time 2 min, oilseed moisture 6 %, cold-pressing temperature 65 °C, cold-pressing time fixed at 40 min.

#### 3.2.2 Optimization of response surface methodology

Response Surface Methodology (RSM), a mathematical-statistical method for analyzing multi-variable interactions, was used (Guan et al., 2022). Based on single-factor experiments, it explored effects of X1 (microwave time), X2 (cold-pressing temperature), X3 (oilseed moisture) on Y (OY).

$$Y=19.60-1.29X_{1}+0.21X_{2}+0.36X_{3}-0.035X_{1}X_{2}-0.43X_{1}X_{3}-0.20X_{2}X_{3}$$

(7) $$-1.64X_{1}^{2}-0.25X_{2}^{2}-0.52X_{3}^{2}$$

In RSM optimization, the OY of parameter combinations ranged from 16.04 to 19.94 % (Table S3), with good fit between experimental and predicted values (Fig. S1). Variance regression analysis of the model was done based on RSM design/results (Table S4). In the model, larger regression coefficients and smaller *p*-values indicate a greater effect on the corresponding response variable. ANOVA results showed that among the models of linear, two-factor interaction (2FI), and quadratic, the quadratic model was highly significant and best fit the experimental data (F = 42.36; *p* < 0.0001) (Table S4). The R^2^ of the quadratic model was 0.9820, indicating that 98.2 % of the variation in response values was related to the variables selected. The adjusted R^2^ (0.9588) agreed well with the predicted R^2^ (0.8277), and the discrepancy between them is less than 0.2, which meant that they fluctuate within a reasonable range. Also, an acceptable signal-to-noise ratio (Adeq Precision = 16.649) and non-significant (*p* = 0.3119) lack of fit are shown for the quadratic model, implying that the model can be used at low error probabilities. Based on F-values, parameter influence on microwave-assisted hybrid cold-pressing: microwave time > moisture content > cold-pressing temperature (Table S4). Overall, the regression model fits the experiments well and can be used for analyzing and predicting experimental results.

Further, results also showed that microwave treatment time, both as linear and quadratic terms, was highly significant (X_1_, *p* < 0.0001; X_1_^2^, *p* < 0.0001), followed by quadratic and linear terms for oilseeds moisture content (X_3_^2^, *p* = 0.0060; X_3_, *p* = 0.0079). In addition, the interaction effect of microwave treatment time and oilseeds moisture content was also significant (X_1_X_3_, *p* = 0.0166). However, both the linear and quadratic terms of the press temperature were not significant (X_2_, *p* = 0.0687; X_2_^2^ *p* = 0.1108). Similarly, the interactions of press temperature and oilseeds moisture content (X_2_X_3_, *p* = 0.1911) and microwave treatment time and press temperature (X_1_X_2_, *p* = 0.8073) were also insignificant within the experimental range. Additionally, the interaction mechanism of microwave treatment time and oilseeds moisture content is mainly that the vibration of polar substances in oilseeds, especially water molecules, will be enhanced after microwave treatment, thereby increasing oil yield. Next, 3D response surface plots showed that for pairwise interactions of the three variables, with one fixed, oil extraction rate rose to a critical point then declined as the other two increased (Fig. S2). At a fixed pressing temperature of 65 °C, the oil yield was positively correlated with microwave treatment time and oilseed moisture content when they were increased to approximately 2 min and 6 %, respectively; however, a further increase in both parameters led to a moderate reduction of the oil yield (Fig. S2A). Furthermore, oil yield improved with increasing pressing temperature in the experimental ranges of microwave treatment time and oilseeds moisture content, but this improvement was not apparent after the pressing temperature exceeded about 66 °C. Therefore, the optimized microwave-assisted hybrid cold-pressing conditions are: microwave treatment time of 1.5 min at 800 W, press temperature of 64 °C, oilseed moisture content of 6.7 %, and the model predicted a maximum oil yield of 19.95 %. Under these conditions, the experimental oil yield was 19.83 ± 0.21 %, which was closely aligned with the predicted value, suggesting that the model developed was statistically dependable.

### 3.3 Physicochemical parameters of oils

**Fig. 3:**
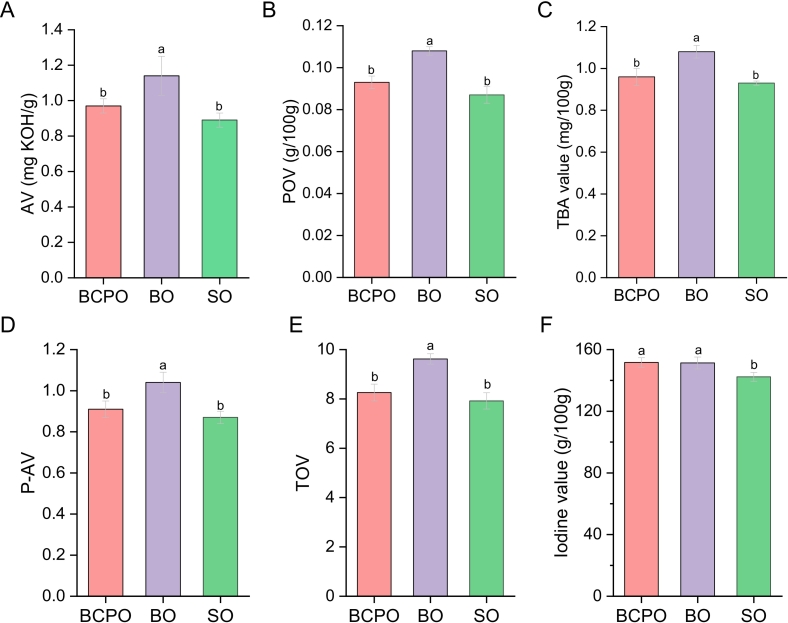
Determination of basic physicochemical properties. A, B, C, D, E, and F respectively represented the determination results of acid value (AV), peroxide value (POV), 2-thiobarbituric acid value (TBA), p-anisidine value (P-AV), total oxidation value (TOV), and iodine value in the oil sample. BCPO, blended cold pressed oil; BO, blended oil; SO, safflower oil. Values and error bars represented the means and standard deviations of duplicated experiments (n = 3), respectively. Different letters in the figure indicated significant differences (p ≤ 0.05).

Fats and oils are prone to oxidation or hydrolysis under light, oxygen, heat and other factors, breaking neutral lipids into glycerol and free FAs, generating unpleasant odors and severely impairing their sensory quality (Xu et al., 2025). It was found that BCPO showed significantly lower primary oxidation levels (AV, POV), secondary oxidation levels (TBA, P-AV), and comprehensive oxidation level (total oxidation value) than BO (*p* < 0.05), though no significant difference in iodine value indicated similar unsaturation degrees between the two (Fig. 3). BCPO had no significant oxidation difference from SO but significantly higher unsaturation than SO (Fig. 3A, B, C, D and F). Thus, compared with the traditional cold-pressed blending process, the microwave-assisted mixed cold-pressing process has significant advantages in delaying oil oxidation without affecting the unsaturation of safflower seed oil blends with balanced PUFAs. Due to the involvement of the FO system with high unsaturation, the microwave-assisted mixed cold-pressing process can improve the unsaturation level of oils to a certain extent compared with the microwave cold-pressing process using a single raw material (SO).

**Fig. 4:**
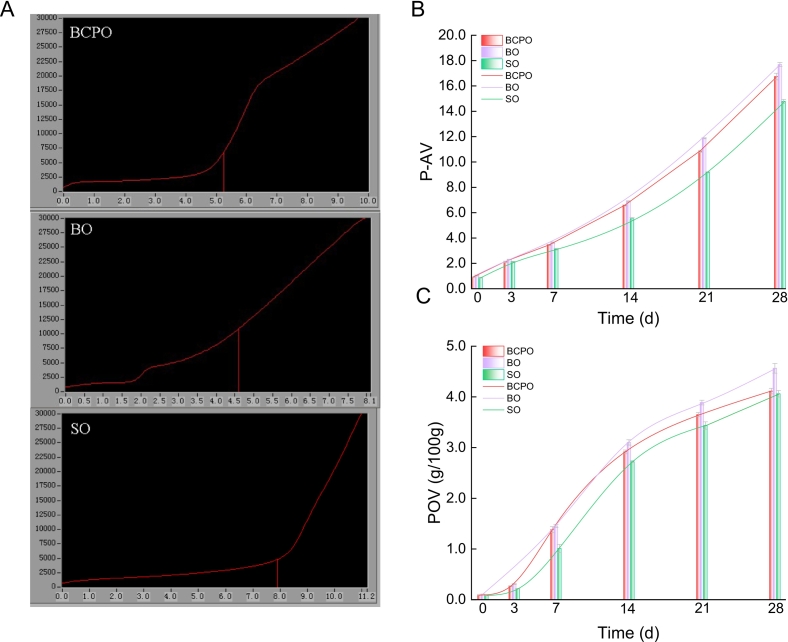
Determination of oxidation stability. A, comparison of oxidation stability index (OSI) values in oil samples. B and C represented the changes in p-AV and POV of oil samples during accelerated oxidation. BCPO, blended cold pressed oil; BO, blended oil; SO, safflower oil. Values and error bars represented the means and standard deviations of duplicated experiments (n = 3), respectively.

Two main methods evaluate oil oxidative stability: Rancimat accelerated oxidation and Schaal oven method. The oxidation stability index (OSI value) is a key indicator of oil oxidative stability; the higher the OSI value, the better the oxidative stability (Zhang et al., 2025). The Rancimat accelerated oxidation method showed that BCPO had an OSI of 5.25 h, longer than BO's 4.60 h, possibly due to its processing technology avoiding the loss of antioxidant substances, enhancing oxidation stability (Fig. 4A). SO had the highest OSI at 7.90 h, likely due to differences in unsaturation and ALA content from the other two oils. Based on the free radical reaction mechanism of lipid autoxidation, the higher the degree of unsaturation of oils, the weaker their oxidation stability, which is consistent with the results of iodine value determination (Fig. 3F) (Zhang et al., 2022). During Schaal oven-accelerated oxidation, POV and P-AV of all oil samples increased (Fig. 4B and C). SO had lower primary and secondary oxidation products at all stages than the other two oils, likely due to its lower ALA content and unsaturation (Fig. 1B, Fig. 3F). BCPO and BO showed similar POV/P-AV trends, but BCPO had lower oxidation products than BO at each stage. In 0–3 d, both had slow POV growth, possibly due to high natural VE (tocopherols) (Fig. 4C). From 3 to 14 d, with VE fully oxidized, POV rose faster and P-AV slower, indicating initial peroxide accumulation. In 14–28 d, BCPO and BO saw faster P-AV growth and slower POV rise, showing enhanced peroxide decomposition and rapid secondary product accumulation. Overall, the oil produced by microwave-assisted mixed cold pressing using safflower seeds as raw material and flaxseeds as ALA-regulating adjunct has better oxidation stability than that from traditional cold-pressed blending process.

### 3.4 FA composition, tocopherols and phytosterols of oils

**Fig. 5:**
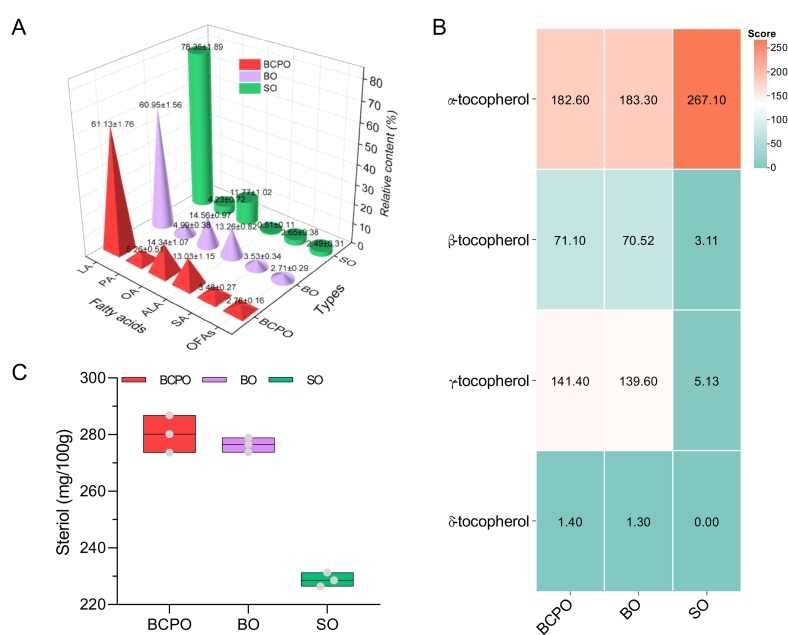
Analysis of nutritional indexes. A, B and C represented the value of fatty acids, tocopherol and phytosterol in oil samples. BCPO, blended cold pressed oil; BO, blended oil; SO, safflower oil. Values and error bars represented the means and standard deviations of duplicated experiments (n = 3), respectively.

FA composition, tocopherols and phytosterols are key indicators for evaluating the nutritional value of vegetable oils (Feng et al., 2022). PUFAs are crucial for human immune regulation and lipid metabolism; tocopherols act as antioxidants, anti-aging and maintain reproductive function; phytosterols help reduce cholesterol, inhibit its intestinal absorption and lower cardiovascular disease risk. In each oil sample, LA, ALA, OA, SA and PA account for over 97 % of total FAs, with other FAs (OFAs) at 2–3 % (Fig. 5A). SO had the highest LA (78.35 ± 1.89 %) and lowest ALA (0.51 ± 0.11 %), with its n-6/n-3 PUFAs ratio far exceeding the recommended dietary intake. However, with linseed oil added (BCPO and BO), ALA increased significantly and the ratio met the standard, showing linseed oil is an effective ALA dietary supplement. BCPO and BO had similar main FAs contents, with over 85 % unsaturated FAs (including >73 % PUFAs), consistent with iodine value results (Fig. 5A, Fig. 3F). Their n-6/n-3 PUFAs ratios (4.69:1 and 4.59:1) met recommended intake, showing microwave-assisted cold-pressed PUFA-balanced safflower blended oil can replace traditional cold-pressed versions in FAs nutrition.

Naturally occurring VE exists in four forms: α-, β-, γ- and δ-tocopherol. In BCPO, α-tocopherol was highest (182.6 ± 4.6 mg/kg), followed by γ- (141.4 ± 3.2 mg/kg), β- (71.1 ± 1.3 mg/kg, ∼half γ-), and δ-tocopherol (1.4 ± 0.12 mg/kg) (Fig. 5B). No significant difference in the four tocopherols between BCPO and BO, showing microwave-assisted cold-pressed oil can replace traditional cold-pressed versions in tocopherol nutrition. Compared with SO, BCPO and BO had increased β-, γ- and δ-tocopherol, especially γ-tocopherol. This is due to high α-tocopherol in SO and FO being rich in γ- and β-tocopherol (main VE components). Studies have shown that daily intake of 2–3 g phytosterols can reduce blood LDL cholesterol by ∼10 % (Gao et al., 2023). SO had the lowest phytosterol content (228.8 ± 2.0 mg/100 g) (Fig. 5C). After adding FO via microwave-assisted mixed pressing or traditional blending, the phytosterol content in the oil samples increased significantly (*p* < 0.05), indicating that with the involvement of FO, its advantage in sterol content can be utilized to enhance the phytosterol level of SO. There was no significant difference in phytosterol content between BCPO and BO (*p* > 0.05), with contents of 280.2 ± 5.4 mg/100 g and 276.4 ± 2.1 mg/100 g respectively.

### 3.5 Volatile aroma profiles of oils

**Fig. 6:**
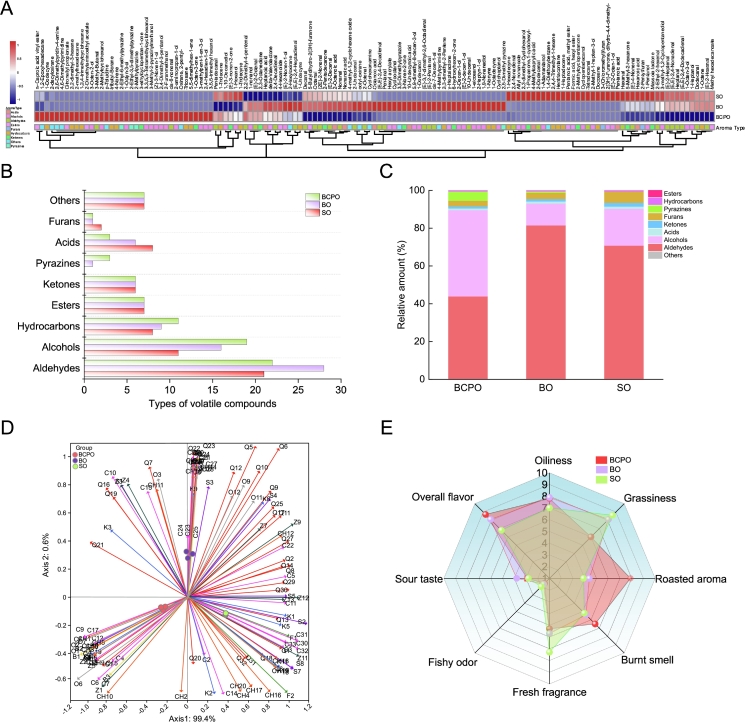
Characterization of aroma compound profiles and senses assessment in Oils. A, the clustering heatmap of aroma compounds; B, types of volatile compounds; C, proportion of various volatile compounds; D, PCA for aroma compounds in oils; The numbering information was detailed in the first and second columns of Table 1. E, sensory evaluation analysis. BCPO, blended cold pressed oil; BO, blended oil; SO, safflower oil.

A total of 137 volatile compounds were identified in the oil samples (Table 1, Fig. 6A). The most diverse aroma compounds in oils were aldehydes and alcohols, followed by furans, ketones, acids, etc. (Fig. 6B and C). In BCPO, a total of 79 volatile compounds were identified, which comprised 22 aldehydes, 19 alcohols, 11 hydrocarbons, 7 esters, 6 ketones, 3 pyrazines, 3 acids, 1 furan, and 7 others. In BCPO, the volatile substances were mainly aldehydes, alcohols, and pyrazines, accounting for 41.8 %, 43.5 %, and 4.7 % of the total volatile compounds, respectively; however, for BO, the aldehydes accounted for 79.3 %, alcohols for only 11.2 %, and pyrazines for less than 1 % (Fig. 6C). The relative concentration of aldehydes in BCPO was about half of that in BO, with hexanal, (E)-2-heptenal and 3-methyl-butanal being the predominant ones (Fig. 6A, Fig. 6C). Notably, 3-methyl-butanal and cis-6-nonenal were volatile aldehyde components specific to the BCPO. Compared to SO, BCPO had a greater variety of aldehydes, alcohols, pyrazines, and hydrocarbons. Most of the identified substances in SO have been reported (Wang, Chen, et al., 2020), while 11 volatile compounds with relatively high contents (>0.1 μg/g), namely 5-methyl-2-hexanone, (E)-2-decenal, 2-undecenal, hexanoic acid, heptanoic acid, 1-heptanol, (E)-3-nonen-2-one, (E,E)-2,4-nonadienal, (E)-4-nonenal, ethenyl hexanoate and 2-dodecenal, were firstly identified in the present study (Fig. 6A and B). These differences may relate to safflower seed growth conditions, pretreatment methods, pressing temperature, etc. Additionally, pyrazines were not detected in the SO, probably due to the non-involvement of flaxseed in the pressing system.

**Table 1:** Composition and quantification of volatile components in oil samples.

No.	Compound	CAS	Relative concentration (μg/kg)		
			BCPO	BO	SO
**Aldehydes**					
Q1	Hexanal	66–25-1	28,823.9 ± 709.1^c^	34,805.3 ± 1165.5^b^	56,948.4 ± 1942.1^a^
Q2	(E)-2-Heptenal	18,829–55-5	5648.2 ± 198.3^c^	8163.3 ± 67.4^b^	8836.8 ± 173.5^a^
Q3	3-Methyl-Butanal	590–86-3	4439.1 ± 66^a^	ND	ND
Q4	(E,E)-2,4-Decadienal	25,152–84-5	3228.1 ± 247^b^	16,707.9 ± 17,576.4^a^	3778.1 ± 92.1^b^
Q5	2-Octenal	2363-89-5	2099.4 ± 73.2^b^	2597.7 ± 169.9^a^	2326.8 ± 49.2^b^
Q6	Nonanal	124–19-6	1553.4 ± 24.5^c^	2617.5 ± 61.1^a^	2173.9 ± 43.4^b^
Q7	(E,E)-2,4-Heptadienal	4313–03-5	1428.6 ± 81.3^b^	3362.5 ± 58.3^a^	ND
Q8	Heptanal	111–71-7	1096.3 ± 76.3^c^	1891.5 ± 44.1^b^	2150.6 ± 36.3^a^
Q9	Octanal	124–13-0	728.5 ± 0.8^c^	1328.4 ± 0.8^a^	1202.9 ± 0.8^b^
Q10	(E)-2-Decenal	3913-81-3	739.3 ± 15^c^	1515.7 ± 70.2^a^	1239.4 ± 98.9^b^
Q11	(E)-2-Hexenal	6728-26-3	674.8 ± 18.1^b^	1410.5 ± 143.8^a^	1389 ± 18.9^a^
Q12	2-Undecenal	2463-77-6	540.3 ± 26.6^c^	1157.3 ± 25.4^a^	749.9 ± 16.1^b^
Q13	(E)-4-Nonenal	2277-16-9	245.2 ± 17.6^c^	270.9 ± 25.5^ab^	314.4 ± 12.3^a^
Q14	(E,E)-2,4-Nonadienal	5910-87-2	220.7 ± 16.3^b^	316.8 ± 13.7^a^	340.3 ± 13.7^a^
Q15	cis-6-nonenal	2277-19-2	233.1 ± 21.1^a^	ND	ND
Q16	(E)-2-Octenal	2548-87-0	112.3 ± 12.7^a^	128.5 ± 16^a^	ND
Q17	Decanal	112–31-2	86.6 ± 24.3^b^	162.2 ± 8.6^a^	150.2 ± 19.8^a^
Q18	2,4-Nonadienal	6750-03-4	54.1 ± 8.5^a^	55 ± 10.7^a^	77.3 ± 10.8^a^
Q19	2,4-Decadienal	2363-88-4	37.8 ± 2.9^b^	53.8 ± 6.4^a^	ND
Q20	Pentadecanal	2765–11-9	33.5 ± 9.7^a^	24.5 ± 8^a^	35.2 ± 3.7^a^
Q21	2,2-Dimethyl-4-pentenal	5497-67-6	19.2 ± 3.3^a^	15.6 ± 4.6^a^	ND
Q22	Pentanal	110–62-3	2.8 ± 2.7^b^	48,323.1 ± 853.5^a^	ND
Q23	(E)-2-Pentenal	1576-87-0	ND	2146 ± 91.5^a^	ND
Q24	cis-7-Decen-1-al	21,661–97-2	ND	713.9 ± 13.4^a^	ND
Q25	(2E)-2-Nonenal	18,829–56-6	ND	328.8 ± 18.9^a^	258.4 ± 25.3^b^
Q26	10-Undecenal	112–45-8	ND	103.4 ± 6.9^a^	ND
Q27	Dodecanal	112–54-9	ND	50.1 ± 4.3^a^	50.8 ± 7.4^a^
Q28	(E)-3,7-dimethyl-2,6-Octadienal	141–27-5	ND	39.3 ± 10.4^a^	ND
Q29	(E,E)-2,4-Dodecadienal	21,662–16-8	ND	25.7 ± 3.2^b^	35 ± 4.5^a^
Q30	2-Ethyl-2-hexenal	645–62-5	ND	27.9 ± 7.3^b^	46.3 ± 9.4^a^
Q31	2-Dodecenal	4826-62-4	ND	ND	104.7 ± 7.4^a^
Q32	Tetradecanal	124–25-4	ND	ND	11.6 ± 3.1^a^
**Alcohols**					
C1	2-aminopropan-1-ol	78–91-1	35,872 ± 1949.3^a^	ND	ND
C2	1-Hexanol	111–27-3	6728.3 ± 190^b^	5386.9 ± 167.5^c^	7996.3 ± 124.2^a^
C3	1-Pentanol	71–41-0	5977.3 ± 212^c^	7129.4 ± 59.1^b^	10,098.1 ± 444.2^a^
C4	2,4-Hexadien-1-ol	111–28-4	1823.9 ± 49.4^a^	ND	ND
C5	1-Octen-3-ol	3391–86-4	1245.8 ± 84.2^b^	1588 ± 61.1^a^	1719.9 ± 60.5^a^
C6	2-Furanmethanol	98–00-0	936.5 ± 16.7^a^	ND	ND
C7	2,4-Dimethyl-3-pentanol	600–36-2	701.1 ± 39.4^a^	ND	ND
C8	2-Methylcyclohexanol	583–59-5	147.3 ± 4.4^a^	ND	ND
C9	3-Octen-2-ol	76,649–14-4	97.9 ± 10.8^a^	ND	ND
C10	1-nonanol	143–08-8	99.9 ± 27.6^a^	117.2 ± 30.6^a^	ND
C11	3-methyl-1,2-Cyclopentanediol	27,583–37-5	80.4 ± 7.3^c^	128.9 ± 22.2^b^	174.2 ± 12.2^a^
C12	(Z)-3-Nonen-1-ol	10,340–23-5	34.7 ± 12^a^	ND	ND
C13	1-Octyn-3-ol	818–72-4	19.7 ± 1.5^a^	ND	ND
C14	1,10-Decanediol	112–47-0	17.3 ± 3.9^a^	ND	24.8 ± 3.7^a^
C15	(E)-2-Nonen-1-ol	31,502–14-4	14.9 ± 3.7^b^	49.4 ± 8^a^	ND
C16	3-Methyl-2-pyrazinylmethanol	160,818–32-6	15.6 ± 3^a^	ND	ND
C17	3,5,5-trimethyl hexanol	3452-97-9	13.7 ± 4.4^a^	ND	ND
C18	3,3,5,5-Tetramethylcyclohexanol	2650-40-0	5.7 ± 1.9^a^	ND	ND
C19	3-methylpent-1-en-3-ol	918–85-4	1.8 ± 1.4^a^	ND	ND
C20	2-Heptyn-1-ol	1002-36-4	ND	758 ± 44^a^	ND
C21	Cyclooctanol	696–71-9	ND	352.8 ± 41.4^a^	ND
C22	1-Heptanol	111–70-6	ND	333 ± 19.9^a^	376.9 ± 24.4^a^
C23	Cycloheptanol	502–41-0	ND	135.2 ± 14.8^a^	ND
C24	1-Dodecanol	112–53-8	ND	71.8 ± 8.8^a^	ND
C25	1-eicosanol	629–96-9	ND	23.6 ± 1.7^a^	ND
C26	(E)-2-decen-1-ol	18,409–18-2	ND	6.8 ± 1.7^a^	ND
C27	5,9-dimethyl-8-decen-3-ol	19,550–54-0	ND	6.2 ± 1.1^a^	ND
C28	1,9-Nonanediol	3937–56-2	ND	5.6 ± 2.3^a^	ND
C29	2-Decen-1-ol	22,104–80-9	ND	1.7 ± 0.2^a^	ND
C30	2-Methyl-1-hepten-3-ol	13,019–19-7	ND	ND	1155.7 ± 108.1^a^
C31	(E)-2-Octen-1-ol	18,409–17-1	ND	ND	315.9 ± 30.2^a^
C32	Cyclopentadecanol	4727-17-7	ND	ND	128.9 ± 20.7^a^
C33	1-Adamantanol	768–95-6	ND	ND	22.4 ± 5.1^a^
C34	2,3-dimethyl-Cyclohexanol	1502-24-5	ND	ND	13.9 ± 3.8^a^
**Acids**					
S1	Heptanoic acid	111-14-8	764.6 ± 6.2^b^	1051.1 ± 56.1^a^	386.7 ± 4.2^c^
S2	Hexanoic acid	142-62-1	281.6 ± 16.1^b^	329.9 ± 38.9^b^	414.7 ± 25.3^a^
S3	Octanoic acid	124-07-2	32.4 ± 2.6^b^	62.4 ± 2.2^a^	37 ± 3.1^b^
S4	Nonanoic acid	112-05-0	ND	130 ± 18.5^a^	80.9 ± 7.2^b^
S5	2-Octenoic acid	1470-50-4	ND	46.5 ± 6.4^a^	30.2 ± 4.8^b^
S6	10-Undecynoic acid	2777-65-3	ND	4.2 ± 0.3^a^	ND
S7	Pentanoic acid	109-52-4	ND	ND	426.8 ± 19.6^a^
S8	4-Methylcaprylic acid	54,947-74-9	ND	ND	2.4 ± 1^a^
**Ketones**					
K1	5-Methyl-2-hexanone	110-12-3	758 ± 21.9^c^	1064.2 ± 86.3^b^	1552.9 ± 105.4^a^
K2	(E)-3-nonen-2-one	18,402-83-0	216.7 ± 11.4^b^	ND	327.1 ± 21.9^a^
K3	2,3-Octanedione	585-25-1	119.4 ± 17.2^a^	136.9 ± 5.2^a^	ND
K4	1-Octen-3-one	4312-99-6	108.8 ± 12.2^a^	ND	ND
K5	Massoia lactone	54,814-64-1	102.4 ± 13.7^b^	125.7 ± 15.3^b^	182.4 ± 12.7^a^
K6	3-ethylcyclopentan-1-one	10,264-55-8	40.4 ± 4.1^a^	ND	ND
K7	3-Nonen-2-one	14,309-57-0	ND	339.3 ± 21.2^a^	ND
K8	2-Pentadecanone	2345-28-0	ND	84.6 ± 3.1^a^	55.7 ± 9^b^
K9	6-hydroxyhexan-2-one	21,856-89-3	ND	28.3 ± 2.8^a^	ND
K10	2-Methylcyclopentanone	1120-72-5	ND	ND	53.3 ± 5.3^a^
K11	1-Propanone,1-cyclohexyl-	1123-86-0	ND	ND	45.2 ± 3.7^b^
**Esters**					
Z1	n-Caproic acid vinyl ester	3050-69-9	613.2 ± 41.1^a^	ND	150.9 ± 20.5^b^
Z2	2-Dimethylaminoethyl acetate	1421-89-2	597.9 ± 47.2^a^	ND	ND
Z3	4-Hexanolide	695-06-7	125.1 ± 8.4^a^	192.8 ± 26.2^b^	ND
Z4	delta-Valerolactone	542-28-9	27.4 ± 1.4^b^	46 ± 2.3^a^	ND
Z5	Citronellyl propionate	141-14-0	20 ± 3.9^a^	ND	ND
Z6	Allyl nonanoate	7493-72-3	15.5 ± 3.2^b^	15.4 ± 3.9^b^	39.1 ± 3.6^a^
Z7	Methyl hexadecanoate	112-39-0	12.7 ± 1.4^b^	24.8 ± 2.9^a^	24.5 ± 3.5^a^
Z8	Hexyl acrylate	2499-95-8	ND	841.6 ± 41.1^a^	ND
Z9	5-Butyldihydro-2(3H)-furanone	104-50-7	ND	158.1 ± 8.9^a^	126.9 ± 8.9^b^
Z10	γ-Heptalactone	105-21-5	ND	74.3 ± 4^a^	ND
Z11	Formic acid, hexyl ester	629-33-4	ND	ND	74.3 ± 3.6^a^
Z12	2(3H)-Furanone, dihydro-4,4-dimethyl-	13,861-97-7	ND	ND	53.7 ± 13.7^a^
Z13	Pentanoic acid, methyl ester	624-24-8	ND	ND	18.7 ± 3.1^a^
**Furans**					
F1	2-Pentylfuran	3777-69-3	3536.2 ± 262.8^c^	4573.5 ± 170.8^b^	6749.5 ± 129.6^a^
F2	3-(Chloromethyl)furan	14,497-29-1	ND	ND	8.6 ± 0.8^a^
**Pyrazines**					
B1	2-Methylpyrazine	109-08-0	5134.8 ± 100.4^a^	ND	ND
B2	2-Ethyl-3,5-dimethylpyrazine	13,360-65-1	495.2 ± 7.2^a^	ND	ND
B3	2-Ethyl-6-methylpyrazine	13,925-03-6	201 ± 37.4^a^	ND	ND
B4	2,5-Dimethyl pyrazine	123–32-0	ND	972.8 ± 61^a^	ND
**Hydrocarbons**					
CH1	1-Dodecyne	765-03-7	303.6 ± 4.5^a^	34.6 ± 2.8^b^	ND
CH2	1-Tridecene	2437-56-1	86.8 ± 9.9^a^	58.1 ± 5.3^b^	86.2 ± 4.8^a^
CH3	5,5-dimethylhex-1-ene	7116-86-1	77.3 ± 5.9^a^	ND	ND
CH4	1-Tridecyne	26,186-02-7	36.6 ± 4.4^b^	3.8 ± 5.4^c^	77.9 ± 1.8^a^
CH5	1,2,4-trimethylcyclohexane	2234-75-5	32 ± 1.5^a^	ND	ND
CH6	4-Nonene	2198-23-4	29.8 ± 1.8^a^	ND	ND
CH7	2,2-dimethyl-3-hexene	3123-93-1	14.1 ± 1.3^a^	ND	ND
CH8	Tricosane	638-67-5	14.2 ± 3^a^	ND	ND
CH9	2-methylhexadecane	1560-92-5	9.6 ± 0.3^a^	ND	ND
CH10	tributyl-Borane	122-56-5	12.1 ± 2.8^a^	ND	ND
CH11	1-Undecyne	2243-98-3	5.9 ± 2.6^b^	16.1 ± 2.5^a^	ND
CH12	1-Octene	111-66-0	ND	269.9 ± 4^a^	266.2 ± 9.7^a^
CH13	2,3-dimethyl-2-Pentene	10,574-37-5	ND	257.8 ± 9.2^a^	ND
CH14	1-Octadecyne	629-89-0	ND	59.6 ± 5.1^a^	ND
CH15	1-Nonyne	3452-09-3	ND	12 ± 2^a^	ND
CH16	1-Hexadecyne	629-74-3	ND	6.3 ± 0.3^b^	58.1 ± 6.2^a^
CH17	1-Heptadecene	6765-39-5	ND	ND	74.6 ± 3.5^a^
CH18	Hentriacontane	630-04-6	ND	ND	13.6 ± 3^a^
CH19	2,4,4-Trimethyl-1-hexene	51,174-12-0	ND	ND	5.2 ± 0.5^a^
CH20	Docosane	629–97-0	ND	ND	4.9 ± 0.8^a^
**Others**					
O1	4,6-Dimethylpyrimidine	1558-17-4	3597.6 ± 123^a^	ND	ND
O2	2,6-Dimethylpyridin-4-amine	3512-80-9	464.7 ± 8.2^a^	ND	ND
O3	2-hexyloxirane	2984–50-1	349.3 ± 21.9^b^	736.8 ± 21.6^a^	209.6 ± 19.3^c^
O4	p-Toluidine	106-49-0	176.8 ± 5.7^a^	ND	ND
O5	2-decyloxirane	2855-19-8	30.6 ± 4.2^a^	1.7 ± 0.8^b^	3.9 ± 0.9^b^
O6	1,2-Epoxyhexadecane	7320-37-8	8.5 ± 1.9^a^	ND	2.3 ± 0.5^b^
O7	Oxirane, 2-pentyl-	5063-65-0	1.7 ± 0.2^a^	ND	ND
O8	4-Methylpyrimidine	3438-46-8	ND	1041.1 ± 40.1^a^	ND
O9	octyl-oxirane	2404-44-6	ND	748.8 ± 30.1^a^	247.6 ± 5.5^b^
O10	3,5-Dimethylpyrazole	67–51-6	ND	50.8 ± 1.8^a^	ND
O11	4-methyl-1,2-cyclohexene oxide	36,099-51-1	ND	43.4 ± 4.5^a^	26.3 ± 1.2^b^
O12	Cyclohexanamine	108–91-8	ND	19 ± 0.8^a^	8.1 ± 0.6^b^
O13	1,4-Diethoxybenzene	122–95-2	ND	ND	168.4 ± 14.3^a^

ND: not detected. Values with different letters for numbers are significantly different (*p* < 0.05).

Aldehydes, especially aliphatic aldehydes, contribute considerably to the flavor of oils, often manifesting as fresh or fatty flavors (Tian et al., 2021). There are two main ways for the production of aldehydes in edible oils: one is the autoxidation of oils during the production and storage process, and the other is the lipid oxygenase pathway triggered by cell fragmentation of oilseeds during pressing or other processes (Dun et al., 2019). Oxidation breaks unsaturated bonds in oils to form hydroperoxides, which further decompose into aldehydes and ketones. Previous studies have found that (E, E)-2,4-decadienal and hexanal are produced by the oxidation of LA, whereas octanal and nonanal are derivatives of OA (Ho et al., 2007), and these findings also explain the relatively high levels of these compounds in BCPO. In addition, the primary aroma-active compounds identified in flaxseed oil are hexanal and nonanal, which primarily contribute to flavors that are fatty, sweet, and nutty (Krist et al., 2006). Alcohols were not only more diverse in the BCPO, but their relative concentration was more than three times higher than that in the BO (Fig. 6B and C). 2-Aminopropan-1-ol, 1-hexanol, and 1-pentanol were the major alcohol compounds (Fig. 6A). Most of the alcohols in oils were reported as important contributors to flavor, mainly presenting grassy and floral aromas, such as 1-octen-3-ol with lavender, rose and hay aromas (Mildner-Szkudlarz & Jeleń, 2008). Regarding pyrazines, three types of pyrazines, 2-methylpyrazine, 2-ethyl-3,5-dimethylpyrazine, and 2-ethyl-6-methyl-pyrazine, were detected in BCPO, while only 2,5-dimethyl pyrazine was detected in the BO. The higher amount of pyrazines in the BCPO could be attributed to the increased mutual friction because of the shape differences between flaxseed and safflower seed during the BCP process, and the increase in local temperature of the oilseeds triggered Maillard reactions, Strecker and pyrolysis of amino acids, which promoted the production of pyrazines (Wei et al., 2015). Pyrazines are commonly considered to be an essential source of roasted flavor in oils (Baker et al., 2003). Additionally, furans are also products of heat treatment and are mainly generated by the thermal breakdown of sugars like glucose and fructose. 2-Pentylfuran was detected in both BCPO and BO, which was responsible for the metallic, green bean, and vegetable flavors (Hao et al., 2020). Esters, ketones, and acids were not much different in content between BCPO and BO, all around 1 %. Esters, primarily produced through the reaction between free FAs and alcohols, contribute to the sweet and fruity fragrance of oils, such as methyl hexadecanoate. In BCPO the major esters were n-caproic acid vinyl ester and 2-dimethylaminoethyl acetate, for BO it was mainly hexyl acrylate. Acids are generally associated with sour or pungent sensations, with heptanoic acid and hexanoic acid being the primary acids in BCPO and BO. Ketones are generally desirable in flavor (Wei et al., 2015), and in BCPO and BO, the ketones were mainly 5-methyl-2-hexanone. Hydrocarbons and other components are not usually considered as important factors for the flavor of fats, oils, and lipid-containing foods due to their high odor thresholds (Wei et al., 2015).

Later, PCA was employed to further exhibit the distinctions in the aroma profiles of the oils. PC1 explained 99.4 % of the variance and PC2 explained 0.6 % of the variance, amounting to 100 % of the total variance (Fig. 6D). A clear separation of BCPO, BO and SO could be recognized. BCPO and BO were clearly separated, indicating differing volatile components between mixed cold pressing and post-cold-pressing mixing. Moreover, 3-Octen-2-ol (C9), (Z)-3-Nonen-1-ol (C12), 1-Dodecyne (CH1), 2-Ethyl-3,5-dimethylpyrazine (B2), etc., showed a strong positive correlation with BCPO; Pentanal (Q22), (E)-2-Pentenal (Q23), 6-hydroxyhexan-2-one (K9), 2-Heptyn-1-ol (C20), etc., were strongly positively correlated with BO samples; and 1-Adamantanol (C33), 2-Pentylfuran (F1), 2-Methyl-1-hepten-3-ol (C30), Hexanal (Q1), 1-Pentanol (C3), etc., had a strong positive correlation with SO, and these results were consistent with those of the heatmap (Fig. 6D and A). Overall, microwave-assisted mixed cold-pressing enhances oil flavor more than microwave cold-pressing with a single oilseed.

### 3.6 Odor activity values (OAV) of aromatic compounds

In general, volatile compounds' contribution to overall aroma depends on their concentration and odor threshold. To identify key aroma substances in oil samples, those with OAV ≥ 1 are considered key, with higher OAV values indicating greater contribution. Results showed that 17, 15, and 13 aromatic compounds with OAV ≥ 1 in BCPO, BO and SO, respectively, of which 13 compounds were identified in all oil samples, including (E)-2-heptenal, 2-pentylfuran, hexanal, (E,E)-2,4nonadienal, 1-hexanol, (E,E)-2,4-decadienal, 1-pentanol, octanal, heptanoic acid, heptanal, 2-octenal, nonanal, and 1-octen-3-ol (Table 2, Fig. S3). Among all the oil samples, (E)-2-heptenal had the highest OAVs in the range of 434.5–679.8, contributing the most to herbaceous and oily odors, followed by 2-pentylfuran with OAVs ranging from 353.6 to 675. Hexanal and (E,E)-2,4-nonadienal also had high OAVs (all>100) (Table 2). Interestingly, BCPO had the largest variety of key aroma components, with 1-octen-3-one, 2-ethyl-3,5-dimethylpyrazine, and 2-ethyl-6-methyl-pyrazine being the unique key aroma components, which also indicated a distinct advantage in BCPO in terms of roast and vegetable flavors. Therefore, the blended cold press process showed greater advantage in volatile aroma contribution than single-material cold pressing.

**Table 2:** Threshold and odor description of the key aromatic compounds in BCPO, BO and SO.

No.	Compound	OAVs			Odor threshold (μg/kg)	Odor description
		BCPO	BO	SO		
1	(E)-2-Heptenal	434.5	627.9	679.8	13	herbaceous, oily
2	2-Pentylfuran	353.6	457.3	675	100	green, bean, metallic
3	Hexanal	221.7	267.7	438.1	130	oily, floral, fatty
4	(E, E)-2,4-Nonadienal	147.1	211.2	226.9	1.5	fatty, waxy
5	(E)-2-Octenal	28.1	32.1	ND	4	vegetable
6	1-Hexanol	16.8	13.5	20.0	400	green, fruity
7	(E, E)-2,4-Decadienal	17.9	92.8	21.0	180	fatty, chicken
8	1-Pentanol	12.7	15.2	21.5	470	sweet, fruity
9	Octanal	12.1	22.1	20.0	60	fatty, honey, citrus
10	1-Octen-3-one	9.9	ND	ND	11	vegetable
11	2-Ethyl-3,5-dimethylpyrazine	7.6	ND	ND	65	roasted, nut
12	Heptanoic acid	7.6	10.5	3.9	100	waxy, cheesy, fruity
13	2-Ethyl-6-methyl- pyrazine	5.0	ND	ND	40	roasted
14	Heptanal	4.4	7.5	8.6	250	nutty, fatty
15	2-Octenal	4.2	5.2	4.7	500	Fatty, green herbal
16	Nonanal	1.5	2.6	2.2	1000	plastic, peel-like
17	1-Octen-3-ol	1.4	1.8	1.9	900	lavender, rose, hay
18	Pentanal	<1	203.5	ND	240	powerful, pungen

ND: not detected.

### 3.7 Sensory evaluation

Sensory evaluation results showed that BCPO scored the highest in burnt flavor (6.5), roasted aroma (7.9), and overall flavor (8.7); SO ranked top in grassy flavor (8.6), fresh aroma (7.3), and fishiness (2.0); BO led in oiliness (7.9) and sourness (3.8) (Fig. 6E). BCPO's oiliness score (7.8) was close to BO's (7.9), while SO had the lowest (7.0), indicating that the addition of FO could enhance the oiliness of SO to varying degrees. SO and BO scored relatively high in grassy flavor (8.6 and 8.1, respectively), whereas BCPO scored lower (6.0)—suggesting that microwave-assisted mixed cold pressing can alter the grassy flavor of SO to some extent. For fresh aroma, SO scored the highest (7.3), followed by BO (5.7) and BCPO (5.3) (Fig. 6E). BCPO's scores for roasted aroma and burnt flavor were significantly higher than others, indicating these are its main features. These results were consistent with GC–MS analysis (Section 3.5) and OAV screening (Section 3.6).

## 4 Conclusion

This study prepared a balanced PUFAs SO blend and comprehensively evaluated its chemical, sensory and nutritional properties. Results showed that the optimal oil extraction efficiency was achieved when the blend mass ratio of safflower seeds to flaxseeds was 1.66–2.60:1, with a pressing temperature of 64 °C, oilseed moisture content of 6.7 %, and microwave treatment at 800 watts for 1.5 min. Subsequently, results also showed microwave-assisted mixed cold pressing delayed oil oxidation, slightly increased unsaturation, and had better oxidative stability than traditional mixed cold pressing, and enhanced the nutritional functionality of the resulting BCPO. Additionally, BCPO was enriched with more aroma components, mainly alcohols, pyrazines and hydrocarbons, such as (E)-2-heptenal, 2-pentylfuran, 1-octen-3-one, 2-ethyl-3,5-dimethylpyrazine and 2-ethyl-6-methylpyrazine. Thus, this study helps produce edible oils with more reasonable PUFA ratios and provides theoretical support for BCP's food industry application.

## CRediT authorship contribution statement

**Xiaochun Zheng:** Writing – original draft, Visualization, Software, Methodology. **Gaoqian Zhang:** Writing – original draft, Methodology, Investigation, Data curation, Conceptualization. **Kejun Wei:** Investigation, Conceptualization. **Hongbin Wu:** Supervision, Resources, Conceptualization. **Jinhu Tian:** Resources, Investigation, Conceptualization. **Xinwen Xu:** Supervision, Resources. **Wenyu Liu:** Writing – review & editing, Methodology, Conceptualization. **Min Liu:** Project administration, Funding acquisition. **Changqing Wei:** Writing – review & editing, Project administration, Methodology, Investigation, Funding acquisition.

## Declaration of competing interest

The authors declare that they have no known competing financial interests or personal relationships that could have appeared to influence the work reported in this paper.

## Data Availability

The authors do not have permission to share data.

## Appendix A Supplementary data

Supplementary material **Figure S1** Comparison of experimental and predicted values. **Figure S2** Effect of interaction between microwave assisted blended cold pressed parameters on oil yield. A, X1X2; B, X1X3; C, X2X3. X1, X2 and X3 represented microwave time, cold-pressing temperature, and oilseed moisture, respectively. **Figure S3** The amount of various key pieces of aroma compounds in oils. BCPO, blended cold pressed oil; BO, blended oil; SO, safflower oil. **Table S1** Standard curves and correlation coefficients of fatty acids, tocopherols and phytosteriol. **Table S2** Independent variables and their coded levels used in Box-Behnken experimental design. **Table S3** Experimental values for BCPO by Box-Behnken experimental design (BBD). **Table S4** Results of the ANOVA for the response surface quadratic model. C.V.%, coefficient of variation; df, degrees of freedom. Supplementary material
