# Supplementary material for: Preparation and study of chemical, sensory, and nutritional values of balanced polyunsaturated fatty acid safflower seed oil blended oil
Source: Food Chem X. 2025 Oct 17;31:103187. doi: 10.1016/j.fochx.2025.103187 (PMC12590036; doi:10.1016/j.fochx.2025.103187)
Supplement: Supplementary material — Figure S1 Comparison of experimental and predicted values. Figure S2 Effect of interaction between microwave assisted blended cold pressed parameters on oil yield. A, X1X2; B, X1X3; C, X2X3. X1, X2 and X3 represented microwave time, cold-pressing temperature, and oilseed moisture, respectively. Figure S3 The amount of various key pieces of aroma compounds in oils. BCPO, blended cold pressed oil; BO, blended oil; SO, safflower oil. Table S1 Standard curves and correlation coefficients of fatty acids, tocopherols and phytosteriol. Table S2 Independent variables and their coded levels used in Box-Behnken experimental design. Table S3 Experimental values for BCPO by Box-Behnken experimental design (BBD). Table S4 Results of the ANOVA for the response surface quadratic model. C.V.%, coefficient of variation; df, degrees of freedom. [file mmc1.docx]

**Supplementary Information for**

**Preparation and study of chemical, sensory, and nutritional values of balanced polyunsaturated fatty acid safflower seed oil blended oil**

Xiaochun Zheng ^b, c, d, 1^, Gaoqian Zhang ^b, c, d, e, 1^, Kejun Wei ^b, c, d^, Hongbin Wu ^a, *^, Jinhu Tian ^f^, Xinwen Xu ^g^, Wenyu Liu ^b, c, d^, Min Liu ^h, i, *^, Changqing Wei ^b, c, d, *^

^1^ These authors equally contributed to this study.

^a^ Xinjiang Academy of Agricultural and Reclamation Science, Xinjiang Uygur Autonomous Region, P. R. China.

^b^ Key Laboratory of Agricultural Product Processing and Quality Control of Specialty (Co-construction by Ministry and Province), School of Food Science and Technology, Shihezi University, Shihezi 832000, Xinjiang Uygur Autonomous Region, P. R. China.

^c^ Nutrition and Safety Control of Xinjiang Production and Construction Corps, School of Food Science and Technology, Shihezi University, Shihezi 832000, Xinjiang Uygur Autonomous Region, P. R. China.

^d^ Engineering Research Center of Storage and Processing of Xinjiang Characteristic Fruits and Vegetables, Ministry of Education, School of Food Science and Technology, Shihezi University, Shihezi 832000, Xinjiang Uygur Autonomous Region, P. R. China.

^e^ Binzhou Zhongyu Food Company Limited Key Laboratory of Wheat Processing, Ministry of Agriculture and Rural Affairs National Industry Technical Innovation Center for Wheat Processing, Shandong Province 256600, China.

^f^ Cooperation Base of Health Food Manufacturing and Quality Control, Zhejiang University, Hangzhou 310058, Zhejiang Province, P. R. China.

^g^ Ili Yaqina Agricultural Development Co. Ili Kazakh autonomous prefecture in Xinjiang 835000, Xinjiang Uygur Autonomous Region, P. R. China.

^h^ Key Laboratory of Xinjiang Phytomedicine Resource and Utilization of Ministry of Education, Shihezi University, Shihezi 832000, Xinjiang Uygur Autonomous Region, P. R. China.

^i^ Institute for Safflower Industry Research of Shihezi University, Shihezi University, Shihezi 832000, Xinjiang Uygur Autonomous Region, P. R. China.

***Corresponding Author:**

Min Liu, School of Pharmacy, Shihezi University, Shihezi 832000, P. R. China, E-mail: liuminshzu@163.com.

Changqing Wei, School of Food Science and Technology/Key Laboratory of Xinjiang Phytomedicine Resource and Utilization of Ministry of Education, Shihezi University, Shihezi 832000, P. R. China, E-mail: [wcq_food@shzu.edu.cn](mailto:wcq_food@shzu.edu.cn).

**Supplementary captions**

**Figures: S1-S3**

**Figure S1** Comparison of experimental and predicted values.

**Figure S2** Effect of interaction between microwave assisted blended cold pressed parameters on oil yield. A, X1X2; B, X1X3; C, X2X3. X1, X2 and X3 represented microwave time, cold-pressing temperature, and oilseed moisture, respectively.

**Figure S3** The amount of various key pieces of aroma compounds in oils. BCPO, blended cold pressed oil; BO, blended oil; SO, safflower oil.

**Tables: S1-S4**

**Table S1** Standard curves and correlation coefficients of fatty acids, tocopherols and phytosteriol.

**Table S2** Independent variables and their coded levels used in Box-Behnken experimental design.

**Table S3** Experimental values for BCPO by Box-Behnken experimental design (BBD).

**Table S4** Results of the ANOVA for the response surface quadratic model. C.V.%, coefficient of variation; df, degrees of freedom.


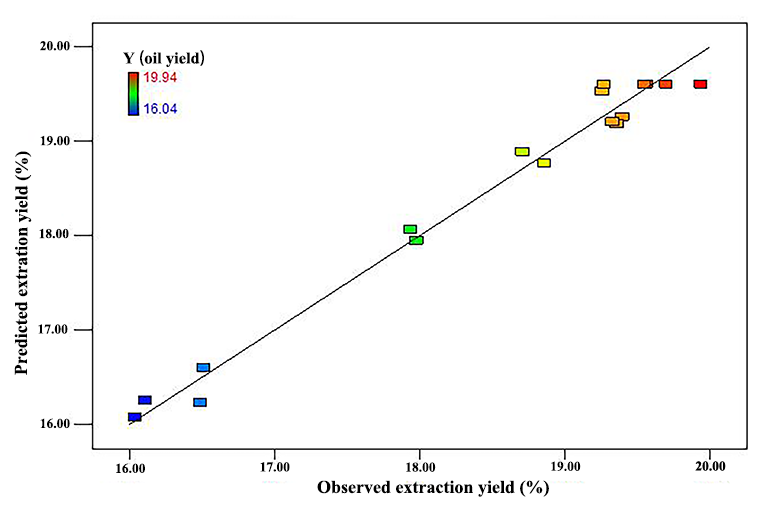


**Figure S1** Comparison of experimental and predicted values.

**
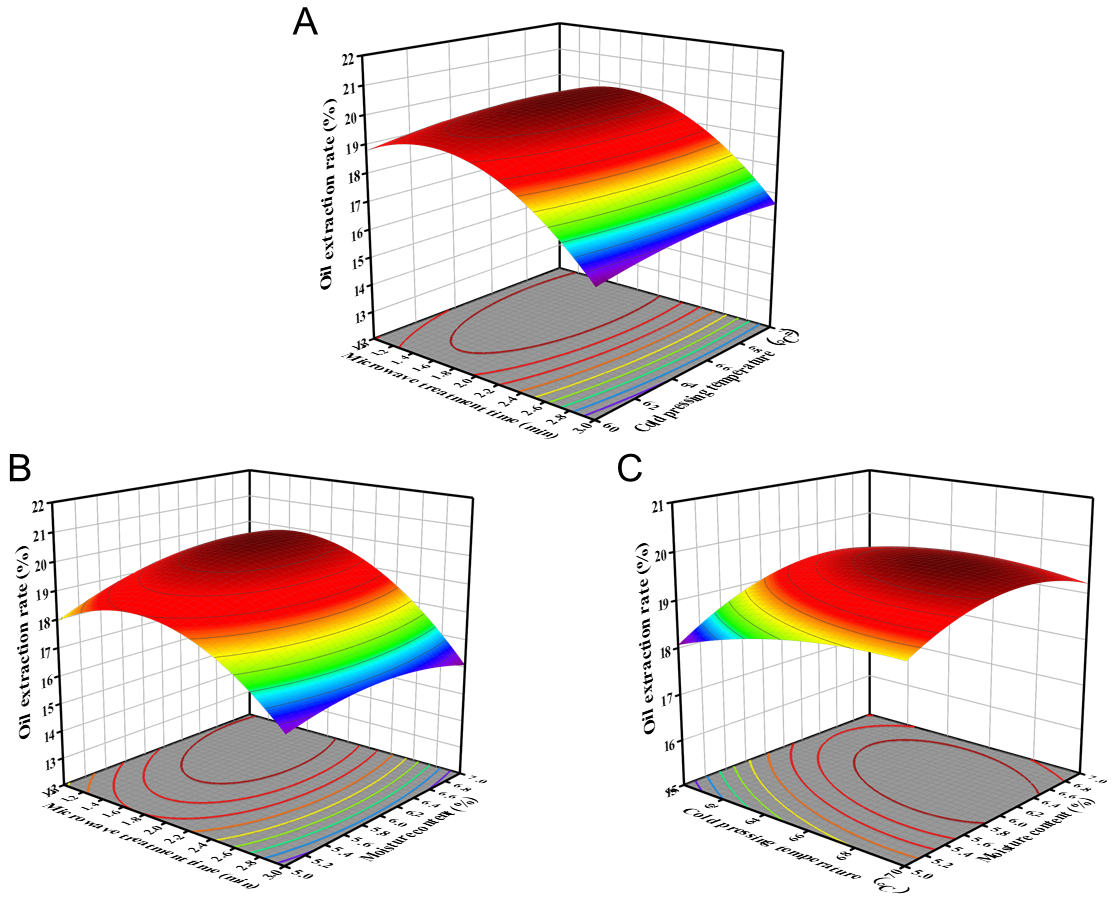
**

**Figure S2** Effect of interaction between microwave assisted blended cold pressed parameters on oil yield. A, X1X2; B, X1X3; C, X2X3. X1, X2 and X3 represented microwave time, cold-pressing temperature, and oilseed moisture, respectively.

**Figure S3** The amount of various key pieces of aroma compounds in oils. BCPO, blended cold pressed oil; BO, blended oil; SO, safflower oil.

**Table S1** Standard curves and correlation coefficients of fatty acids, tocopherols and phytosteriol.

| Fatty acids | Standard curves | R^2^ |
| --- | --- | --- |
| Linoleic acid | $\text{y=6056.6x+45264}$ | 0.9908 |
| α-Linolenic acid | $\text{y=20015x-546853}$ | 0.9919 |
| Oleic acid | $\text{y=5913.4x+5668.5}$ | 0.9901 |
| Palmitic acid | $\text{y=21222x-4357.8}$ | 0.9900 |
| Stearic acid | $\text{y=134074x-3057}$ | 0.9909 |
| α-Tocopherol | y=10147x+1205.9 | 0.991 |
| β-Tocopherol | y=17462x+1355.6 | 0.997 |
| γ-Tocopherol | y=21904x+3983.8 | 0.998 |
| δ-Tocopherol | y=11732x+1472.8 | 0.997 |
| Phytosterol | y=0.0025x-0.0132 | 0.998 |

**Table S2** Independent variables and their coded levels used in Box-Behnken experimental design.

| Independent variables | Codes | coded levels | | |
| --- | --- | --- | --- | --- |
|  |  | Low (-1) | Middle (0) | High (+1) |
| Microwave treatment time (min) | X_1_ | 1 | 2 | 3 |
| Press temperature (℃) | X_2_ | 60 | 65 | 70 |
| Oilseeds moisture content (%) | X_3_ | 5 | 6 | 7 |

**Table S3** Experimental values for BCPO by Box-Behnken experimental design (BBD).

| Run |  | Coded variable | | | |  | Process variable | | | |  | OY (%) | |
| --- | --- | --- | --- | --- | --- | --- | --- | --- | --- | --- | --- | --- | --- |
|  |  | X_1_ | X_2_ | X_3_ |  | | X_1_^a^ | X_2_^b^ | X_3_^c^ |  | | Experimental | Predicted |
| 1 |  | 1 | 0 | 1 |  | | 3 | 65 | 7 |  | | 16.04 | 16.08 |
| 2 |  | 0 | 0 | 0 |  | | 2 | 65 | 6 |  | | 19.55 | 19.60 |
| 3 |  | -1 | 0 | -1 |  | | 1 | 65 | 5 |  | | 17.98 | 17.95 |
| 4 |  | 0 | 1 | 1 |  | | 2 | 70 | 7 |  | | 19.33 | 19.20 |
| 5 |  | 0 | 0 | 0 |  | | 2 | 65 | 6 |  | | 19.56 | 18.18 |
| 6 |  | 0 | 0 | 0 |  | | 2 | 65 | 6 |  | | 19.94 | 19.60 |
| 7 |  | -1 | -1 | 0 |  | | 1 | 60 | 6 |  | | 18.86 | 18.77 |
| 8 |  | 0 | -1 | 1 |  | | 2 | 60 | 7 |  | | 19.36 | 19.18 |
| 9 |  | 0 | 0 | 0 |  | | 2 | 65 | 6 |  | | 19.27 | 19.60 |
| 10 |  | 1 | 1 | 0 |  | | 3 | 70 | 6 |  | | 16.51 | 16.60 |
| 11 |  | 1 | 0 | -1 |  | | 3 | 65 | 5 |  | | 16.49 | 16.22 |
| 12 |  | -1 | 0 | 1 |  | | 1 | 65 | 7 |  | | 19.26 | 19.53 |
| 13 |  | 0 | 0 | 0 |  | | 2 | 65 | 6 |  | | 19.70 | 19.60 |
| 14 |  | -1 | 1 | 0 |  | | 1 | 70 | 6 |  | | 19.40 | 19.26 |
| 15 |  | 0 | 1 | -1 |  | | 2 | 70 | 5 |  | | 18.71 | 18.89 |
| 16 |  | 1 | -1 | 0 |  | | 3 | 60 | 6 |  | | 16.11 | 16.25 |
| 17 |  | 0 | -1 | -1 |  | | 2 | 60 | 5 |  | | 17.94 | 18.01 |

OY, oil yield. ^a^X_1_= Microwave treatment time; ^b^X_2_= Press temperature; ^c^X_3_= Oilseeds moisture content.

**Table S4** Results of the ANOVA for the response surface quadratic model. C.V.%, coefficient of variation; df, degrees of freedom.

| Source | Standard deviation | R^2^ | Adjusted R^2^ | | Predicted R^2^ | |
| --- | --- | --- | --- | --- | --- | --- |
| Linear | 1.07 | 0.4891 | 0.3823 | | 0.1659 | |
| 2FI | 1.18 | 0.5289 | 0.2463 | | 0.5207 | |
| Quadratic | 0.28 | 0.9820 | 0.9588 | | 0.8277 | |
|  |  |  |  |  |  |  |
| Source | Sum of square | Mean square | df | | F-value | *p*-value^a^ |
| Model | 29.12 | 3.24 | 9 | | 42.36 | < 0.0001 |
| X_1_ | 13.39 | 13.39 | 1 | | 175.32 | < 0.0001 |
| X_2_ | 0.35 | 0.35 | 1 | | 4.62 | 0.0687 |
| X_3_ | 1.03 | 1.03 | 1 | | 13.48 | 0.0079 |
| X_1_X_2_ | 4.900E-003 | 4.900E-003 | 1 | | 0.064 | 0.8073 |
| X_1_X_3_ | 0.75 | 0.75 | 1 | | 9.80 | 0.0166 |
| X_2_X_3_ | 0.16 | 0.16 | 1 | | 2.09 | 0.1911 |
| X_1_^2^ | 11.30 | 11.30 | 1 | | 147.95 | < 0.0001 |
| X_2_^2^ | 0.25 | 0.25 | 1 | | 3.33 | 0.1108 |
| X_3_^2^ | 1.15 | 1.15 | 1 | | 15.09 | 0.0060 |
| Residual | 0.53 | 0.076 | 7 | |  |  |
| Lack of fit | 0.30 | 0.099 | 3 | | 1.66 | 0.3119 |
| Pure error | 0.24 | 0.060 | 4 | |  |  |
| Total | 29.66 |  | 16 | |  |  |
| C.V.%=1.50, Adeq Precision=16.649 | | | | | | |

^a^ *p*<0.01 highly significant; 0.01<*p*< 0.05 significant; *p*>0.05 not significant.
